# Selection of reference genes for gene expression studies in pig tissues using SYBR green qPCR

**DOI:** 10.1186/1471-2199-8-67

**Published:** 2007-08-15

**Authors:** Ann-Britt Nygard, Claus B Jørgensen, Susanna Cirera, Merete Fredholm

**Affiliations:** 1 University of Copenhagen, Faculty of Life Sciences, Department of Basic Animal and Veterinary Sciences, Division of Genetics and Bioinformatics, Groennegaardsvej 3, 1870 Frederiksberg C, Denmark

## Abstract

**Background:**

Real-time quantitative PCR (qPCR) is a method for rapid and reliable quantification of mRNA transcription. Internal standards such as reference genes are used to normalise mRNA levels between different samples for an exact comparison of mRNA transcription level. Selection of high quality reference genes is of crucial importance for the interpretation of data generated by real-time qPCR.

**Results:**

In this study nine commonly used reference genes were investigated in 17 different pig tissues using real-time qPCR with SYBR green. The genes included beta-actin (*ACTB*), beta-2-microglobulin (*B2M*), glyceraldehyde-3-phosphate dehydrogenase (*GAPDH*), hydroxymethylbilane synthase (*HMBS*), hypoxanthine phosphoribosyltransferase 1 (*HPRT1*), ribosomal protein L4 (*RPL4*), succinate dehydrogenase complex subunit A (*SDHA*), TATA box binding protein (*TPB*)and tyrosine 3-monooxygenase/tryptophan 5-monooxygenase activation protein zeta polypeptide (*YWHAZ*). The stability of these reference genes in different pig tissues was investigated using the geNorm application. The range of expression stability in the genes analysed was (from the most stable to the least stable): *ACTB*/*RPL4*, *TBP*, *HPRT*, *HMBS*, *YWHAZ*, *SDHA*, *B2M *and *GAPDH*.

**Conclusion:**

Expression stability varies greatly between genes. *ACTB, RPL4*, *TPB *and *HPRT1 *were found to have the highest stability across tissues. Based on both expression stability and expression level, our data suggest that *ACTB *and *RPL4 *are good reference genes for high abundant transcripts while *TPB *and *HPRT1 *are good reference genes for low abundant transcripts in expression studies across different pig tissues.

## Background

Real-time quantitative PCR (qPCR) is an efficient method for quantification of mRNA transcription levels due to its high sensitivity, reproducibility and large dynamic range; in addition, real-time qPCR is fast, easy to use and provides simultaneous measurement of gene expression in many different samples for a limited number of genes. One of the critical steps in comparing transcription profiles is accurate normalisation, therefore, a number of variables should be controlled such as amount of starting material, enzymatic efficiencies, differences in transcriptional activity and presence of inhibitors in different sample materials. One way to standardize samples is to quantify the starting material i.e. number of cells or quantity of RNA. However, this does not take the RNA quality and enzymatic efficiencies into account. To control these variables, which are not a result of the experimental design, normalisation using reference genes are routinely used e.g. [1-3]. The accurate quantification of reference genes allows normalisation of differences in amount of the amplified cDNA in individual samples. The normalisation adjusts for differences in amount and quality of starting material and differences in RNA preparation and cDNA synthesis, since the reference gene is exposed to the same preparation steps as the gene of interest. Recently a set of reference genes (*ACTB*, *TBP *and topoisomerase (DNA) II beta (*TOP2B*)) have proven suitable for normalisation of qPCR data of backfat and longissimus dorsi muscle in the pig [4].

In the present study the expression stability and expression level of nine potential reference genes have been compared in 17 different pig tissues. This has enabled us to assess the suitability of the genes for normalisation of mRNA across several tissues leading to the identification of the best reference genes for studies of high and low abundance transcripts, respectively. The suitable reference genes from this study can be used for normalisation of qPCR data in several tissues. Thus, the study gives useful information of importance for a broad range of different functional studies.

## Results

### Candidate reference genes

Nine housekeeping genes were selected from commonly used reference genes [5-7]. Gene symbols, their full names, functions and pig EST accession numbers are listed in Table 1. Genes with different functions were chosen in order to avoid genes belonging to the same biological pathways that may be co-regulated. The porcine sequences of the genes were obtained by fasta search with the human cDNA sequence for each gene against a porcine EST database[8].

**Table 1:** Selected candidate reference genes

Gene symbol	Gene Name	Accession Number	Function
ACTB	Beta-actin	DQ845171	involved in cell motility, structure and integrity
B2M1	Beta-2-microglobulin	DQ845172	cytoskeletal protein involved in cell locomotion
GAPDH	Glyceraldehyde-3-phosphate dehydrogenase	DQ845173	carbohydrate metabolism
HMBS	Hydroxymethylbilane synthase	DQ845174	heme biosynthesis
HPRT1	Hypoxanthine phosphoribosyltransferase 1	DQ845175	purine ribonulceoside salvage
RPL4	Ribosomal protein L4	DQ845176	Structural constituent of ribosome
SDHA	Succinate dehydrogenase complex, subunit A	DQ845177	Tricarboxylic acid cycle
TBP	TATA box binding protein	DQ845178	transcription initiation from RNA polymerase II promotor
YWHAZ	Tyrosine 3-monooxygenase/tryptophan 5-monooxygenase activation protein, zeta polypeptide	DQ845179	Protein domain specific binding

### QPCR efficiency and variability

Standard curves were generated using relative concentration vs. the threshold cycle (Ct). The linear correlation coefficient (R^2^) of all the nine genes ranged from 0.998 to 1.000. Based on the slopes of the standard curves, the amplification efficiencies of the standards ranged from 93%~103%, (derived from the formula E = 10 ^1/-slope ^-1). This calculation method results in efficiencies higher than 100% which is an overestimate of the "real efficiency" [9]. The not normalised Ct values of all the nine genes in all the samples were within 13.5 to 30.5 cycles, covered by the range of the standard curves.

### Expression level and stability of putative reference genes in various tissues

**Figure 1:**
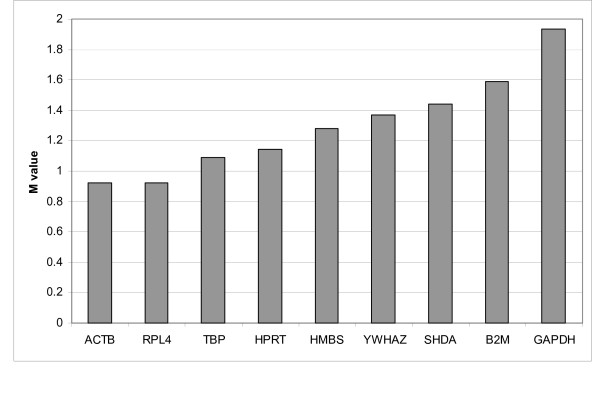
**Gene expression stability of candidate reference genes**. Gene expression stability of candidate reference genes in different pig tissues analyzed by the geNorm program. Threshold for eliminating a gene as unstable was M ≥ 1.5. The respective genes and gene names are: beta-actin (*ACTB*), beta-2-microglobulin (*B2M*), glyceraldehyde-3-phosphate dehydrogenase (*GAPDH*), hydroxymethylbilane synthase (*HMBS*), ribosomal protein L4 (*RPL4*), succinate dehydrogenase complex subunit A (*SDHA*), TATA box binding protein (*TPB*) and tyrosine 3-monooxygenase/tryptophan 5-monooxygenase activation protein zeta polypeptide (*YWHAZ*).

**Figure 2:**
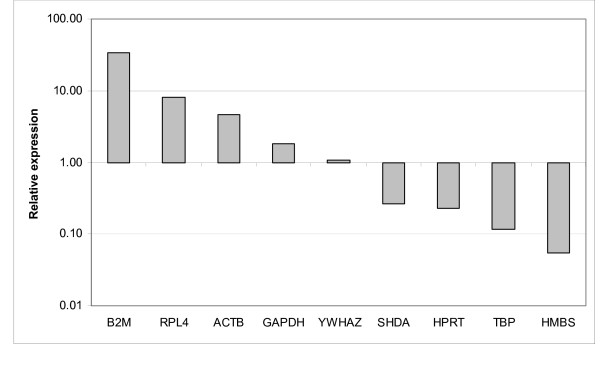
**The relative expression level of the reference candidate genes in pig tissues**. The relative expression level of the reference candidate genes in pig tissues. HMBS was the lowest expressed gene and B2M was the highest expressed gene. The respective genes and gene names are: beta-actin (*ACTB*), beta-2-microglobulin (*B2M*), glyceraldehyde-3-phosphate dehydrogenase (*GAPDH*), hydroxymethylbilane synthase (*HMBS*), ribosomal protein L4 (*RPL4*), succinate dehydrogenase complex subunit A (*SDHA*), TATA box binding protein (*TPB*) and tyrosine 3-monooxygenase/tryptophan 5-monooxygenase activation protein zeta polypeptide (*YWHAZ*).

The range of expression stability calculated by geNorm [6] in the genes analysed was (from the most stable to the least stable): *ACTB*/*RPL4*, *TBP*, *HPRT1*, *HMBS*, *YWHAZ*, *SDHA*, *B2M *and *GAPDH *(Figure 1). The M values for *ACTB*, *RPL4*, *TBP*, *HPRT1*, *HMBS*, *YWHAZ *and *SDHA *were lower than 1.5, and therefore it was concluded that these genes have comparable stability in different pig tissues. For calculation of normalization factor (NF) geNorm suggested the five most stable reference genes. The NF was calculated based on the geometric mean of the Ct values. Normalised Ct values are shown in Table 2. After normalization against the NF, the ranking of the relative expression levels was (from high to low): *B2M*, *RPL4*, *ACTB*, *GAPDH*, *YWHAZ*, *SDHA*, *HPRT1*, *TBP *and *HMBS *(Figure 2). Furthermore, *HMBS*, and *GAPDH *showed tissue-specific regulation, i.e. *HMBS *is clearly up-regulated in bone marrow and *GAPDH *is clearly up-regulated in muscle tissue.

**Table 2:** RNA transcription levels (normalised Ct values) of the candidate reference genes. Values in parentheses shows the ranking of genes in the specific tissue calculated by GeNorm. Values in bold have an M-value above 1.5.

	B2M	RPL4	ACTB	GAPDH	YWHAZ	SHDA	HPRT	TBP	HMBS
muscle	14.7(4)	16.6(**7**)	17.9(**8**)	13.2(5)	20.0(6)	19.5(**9**)	22.5(1)	23.8(1)	24.5(3)
adipose	15.8(-)	17.9(-)	16.5(-)	16.9(-)	17.4(-)	19.9(-)	20.8(-)	21.8(-)	22.8(-)
small intestine	12.8(3)	16.2(1)	16.5(**8**)	17.2(7)	19.4(5)	19.8(**9**)	22.7(4)	23.4(1)	22.8(6)
skin	15.4(5)	16.4(1)	16.7(6)	18.4(4)	18.3(1)	21.8(3)	23.3(**9**)	23.2(8)	24.6(7)
stomach	13.0(6)	17.3(3)	15.5(1)	18.9(**7**)	18.4(4)	21.1(**9**)	23.1(5)	23.7(**8**)	24.0(1)
pancreas	15.2(**6**)	16.2(**8**)	17.1(**7**)	20.6(1)	21.3(5)	21.2(**9**)	22.9(1)	23.7(3)	23.9(4)
kidney	12.9(5)	16.1(4)	15.8(1)	16.6(3)	18.2(7)	18.0(9)	21.2(1)	21.4(6)	22.4(8)
lung	12.5(6)	16.8(1)	15.4(3)	18.8(7)	18.6(1)	21.4(**9**)	21.2(**8**)	22.5(4)	24.0(5)
thymus	14.6(3)	16.3(1)	15.3(8)	17.4(4)	17.5(7)	21.6(9)	22.0(1)	21.8(5)	23.7(6)
hippocampus	13.2(7)	16.2(**9**)	15.8(5)	18.3(4)	18.1(3)	21.0(6)	22.1(**8**)	22.8(1)	24.2(1)
liver	13.2(**9**)	16.8(5)	16.5(4)	17.7(6)	21.0(**7**)	19.8(1)	19.8(3)	23.1(1)	23.3(**8**)
cortex cerebri	15.6(3)	17.1(4)	16.2(1)	16.3(1)	16.9(6)	19.2(**8**)	20.0(**9**)	21.7(5)	23.0(7)
bone marrow	15.1(3)	17.2(**7**)	16.4(**8**)	19.1(5)	19.8(4)	21.9(**6**)	21.6(1)	22.5(1)	19.9(**9**)
cerebellum	16.7(9)	17.2(7)	17.3(4)	17.6(8)	17.9(1)	20.9(6)	20.9(5)	21.8(1)	23.9(3)
lymph nodules	15.3(1)	16.3(5)	16.2(6)	19.2(9)	18.9(3)	20.3(8)	21.3(7)	20.2(4)	23.6(1)
heart	13.9(4)	15.8(1)	16.9(3)	14.6(**8**)	17.6(**6**)	17.1(**7**)	21.2(1)	21.5(5)	22.9(**9**)
bladder	14.9(-)	15.5(-)	16.3(-)	19.2(-)	18.7(-)	21. (-)	21.5(-)	21.5(-)	23.8(-)

## Discussion

For an exact comparison of mRNA transcription in different samples or tissues it is crucial to choose the appropriate reference gene. The optimal reference gene should be constantly transcribed in all types of cells at any time in cell cycle and differentiation. Moreover the transcription of such a gene should not be regulated by internal or external influences, at least not more than the general variation in RNA synthesis. The reference gene used for normalisation of gene expression in real-time qPCR studies should also pass through the same steps of analysis as the gene to be quantified. However, such a perfect reference gene does probably not exist. The stability in expression of often used reference genes such as *GAPDH *and *ACTB *has been shown to vary considerably and are consequently unsuitable as reference genes for normalisation of gene expression analysis in some cases [5,10-12]. Also the low expressed reference gene *TBP *is highly regulated when comparing normal and tumour tissues [13].

Numerous studies have been carried out in order to evaluate reference genes in specific tissues in several species. The majority of these studies are directed towards specific tissues [1,3,7,14]. They clearly demonstrate that it is very difficult to find a 'universal' reference gene having stable expression in all cell types and tissues, and in particular to find reference genes that remain stable between samples taken at different time points under different experimental conditions. The first priority, however, is to identify genes with stable expression preferably across cell types since many real-time qPCR studies are performed on cDNA isolated from tissues with a mixed cell population.

There have been limited experiments of reference gene selection for use in other livestock and production animal species. Bogaert et al. [15] proposed ubiquitin (*UBB*), *ACTB *and *B2M *as reference genes for normalisation of qPCR data for normal equine skin. In cattle studies, De Ketelaere *et al*. [16] selected *SDHA*, *YWHAZ*, and 18S rRNA as being the most stable genes for accurate normalisation of qPCR of bovine polymorphonuclear leukocytes and Goossens *et al*. [2] found *YWHAZ*, *GAPDH *and *SDHA *to be the most stable reference genes across different preimplantation embryonic stages. Common for the bovine studies are the target of the studies, which are single cell populations. Garcia-Crespo *et al*. [17] compared expression of six potential reference genes in sheep tissues and found *GAPDH *with the lowest variation among the panel of six tissues, whereas *ACTB *and *YWHAZ *showed the worst score in variability. This is not in agreement with our findings but can be a result of the tissue composition. At present three studies have examined reference genes in pig i.e. Foss *et al*. [18] describes the use of *GAPDH*, *ACTB *and *HPRT *in immune cells and tissues using northern blot and PCR. In this study *GAPDH *was shown to be more stable than *ACTB*. Erkens et al [4] validated mRNA expression stability of 10 reference genes in porcine backfat and longissimus dorsi muscle. The most stable reference genes suitable for normalisation of qPCR data of backfat and longissimus dorsi muscle in the pig was *ACTB*, *TBP *and *TOP2B*. Kuijk et al. [19] investigated transcription levels of seven frequently used reference genes (inclusive *B2M*, *ACTB*, *GAPDH*) at different stages of early porcine embryonic development. Our study is the first detailed study of the stability and level of pig reference genes across a large number of tissues. Our findings confirm studies demonstrating tissue specific regulation of some of the commonly used housekeeping genes. Furthermore, it provides recommendations for choice of reference genes in studies where high and low abundant transcripts are under investigation. In agreement with the findings of Erkens et al. [4] both *TBP *and *ACTB *are suitable reference genes. However, our data show that *ACTB *is most relevant for high abundant transcripts, while *TPB *is most relevant for low abundant transcripts. It is clear from both Erkens et al. [4] and our study that *GAPDH *is quite unstable and not suitable as a reference gene.

## Conclusion

Our study demonstrates that expression stability varies greatly between genes. Seven of the nine genes investigated (i.e. *ACTB*, *RPL4*, *TBP*, *HPRT1*, *HMBS*, *YWHAZ, SDHA*) were found to have a high stability across tissues. Two of the genes investigated were shown to have tissue-specific regulation, i.e. *HMBS *is clearly up-regulated in bone marrow and *GAPDH *is clearly up-regulated in muscle. Based on both expression stability and expression level, our data suggest that *ACTB *and *RPL4 *are good reference genes for high abundant transcripts while *TPB *and *HPRT1 *are good reference genes for low abundant transcripts in expression studies across different pig tissues.

## Methods

### Candidate reference genes and primer design

Nine housekeeping genes were selected from commonly used reference genes [5-7]. The porcine sequences of the genes were obtained by fasta search with the human cDNA sequence for each gene against a porcine EST database[8]. The consensus sequences were used for comparison with genomic porcine sequences, if available, to reveal the exon-intron structure. When no genomic porcine sequence was available the gene structure was obtained by comparison of the pig sequence with human and mouse genomic sequence assuming that the exon-exon boundaries are conserved between human, mouse and pig.

All the primers were designed by Primer 3 software [20]. For information on primer sequences see Table 3. Primers spanning at least one intron were chosen to minimize inaccuracies due to genomic DNA contamination. If the genes had known pseudogenes, primers were chosen according to the alignment results between the genes and the pseudogenes, so the primers were unique to the genes and different from the pseudogenes. The secondary structure of the amplicons was analyzed by Mfold using the criteria -3<dG<0 [21] to optimize the PCR efficiency. Primers and amplicons were in silico verified with BLASTN for specificity and the size of the PCR products was confirmed with gel electrophoresis.

**Table 3:** Primers, PCR conditions and qPCR efficiency

Gene symbol	Oligo sequence (5'→3')	Amplicon length	Tm (°C)	E%
ACTB	CACGCCATCCTGCGTCTGGA AGCACCGTGTTGGCGTAGAG	100	63	97
B2M1	CAAGATAGTTAAGTGGGATCGAGAC TGGTAACATCAATACGATTTCTGA	161	58	101
GAPDH	ACACTCACTCTTCTACCTTTG CAAATTCATTGTCGTACCAG	90	60	103
HMBS2	AGGATGGGCAACTCTACCTG GATGGTGGCCTGCATAGTCT	83	58	98
HPRT1	GGACTTGAATCATGTTTGTG CAGATGTTTCCAAACTCAAC	91	60	99
RPL4	CAAGAGTAACTACAACCTTC GAACTCTACGATGAATCTTC	122	60	97
SDHA	CTACAAGGGGCAGGTTCTGA AAGACAACGAGGTCCAGGAG	141	58	98
TBP1	AACAGTTCAGTAGTTATGAGCCAGA AGATGTTCTCAAACGCTTCG	153	60	93
YWHAZ	TGATGATAAGAAAGGGATTGTGG GTTCAGCAATGGCTTCATCA	203	60	99

### RNA extraction

Seventeen different porcine tissues were collected from three young female siblings. Total RNA was extracted using different protocols depending of the tissue: TRIreagent^® ^(Molecular Research Centre, inc.) for liver, kidney, thymus, RNeasy lipid kit (Qiagen) for adipose (subcutaneous), cortex cerebri, cerebellum, hippocampus, lymph nodules (jejunal), RNeasy Fibrous Kit (Qiagen) for muscle (longissimus dorsi), heart (muscle), skin (dermis and epidermis) and RNeasy kit (Qiagen) for pancreas, bone marrow, bladder, lung, stomach (mucosal membranes), small intestine (mucosal membranes) according to each manufacturer protocol. Contaminating DNA was degraded by treating each sample with RQ1 RNase-free DNase (Promega) according to the instructions manual, followed by a spin-column purification (Qiagen RNeasy). The total RNA was quantified by optical density and the quality was evaluated by gel electrophoresis. Intact rRNA subunit of 28S and 18S were observed on the gel indicating minimal degradation of the RNA.

### cDNA synthesis

One μg of total RNA was reverse transcribed at 42°C using Improm-II™ reverse trancriptase (Promega) and Oligo(dT) according to the manufacturers recommendations. Prior to use in qPCR cDNA was diluted 1:8 with H_2_O.

### Quantitative PCR with SYBR green

For each transcript a standard curve was constructed using the purified PCR product generated for each specific primer pair. Single reactions were prepared for each cDNA along with each serial of dilution using the Brilliant^® ^SYBR^® ^Green Master Mix (Stratagene). Each PCR reaction also included a reverse transcription negative control (without reverse transcriptase) to confirm the absence of genomic DNA, a non template negative control to check for primer-dimer and a porcine genomic DNA control to verify no specific amplification with the primers. Each reaction consisted of 20 μl containing 2 μl of cDNA and 5 pmol of each primer. The real time qPCR was run on MX3000p (Stratagene). The cycling conditions were 1 cycle of denaturation at 95°C/10 min, followed by 40 three-segment cycles of amplification (95°C/30 sec, 58°C–63°C (gene depending, see table 2)/1 min, 72°C/30 sec) where the fluorescence was automatically measured during PCR and one three-segment cycle of product melting (95°C/1 min, 55°C/30 sec, 95°C/30 sec). The baseline adjustment method of the Mx3000 (Stratagene) software was used to determine the Ct in each reaction. A melting curve was constructed for each primer pair to verify the presence of one gene-specific peak and the absence of primer dimmer. All samples were amplified in duplicates and the mean was used for further analysis.

### Analysis of expression stability

The gene expression levels were measured by real-time qPCR, and the expression stabilities were evaluated by the M value of geNorm [6]. The M value for each reference gene is the average pairwise variation for that gene with all the other tested control genes. Stepwise exclusion of the gene with the highest M value allows ranking of the tested genes according to their expression stability.

## Authors' contributions

ABN has been responsible for the experimental work and made substantial contributions to conception and design of the study. Furthermore, she has been involved in drafting the manuscript. CBJ has made substantial contributions to conception and design of the study and been involved in drafting the manuscript and revising it critically for important intellectual content. SC has made substantial contributions to the experimental work and been involved in drafting the manuscript and revising it critically for important intellectual content. MF has made substantial contributions to conception and design of the study. She has been involved in drafting the manuscript and revising it critically for important intellectual content, and given final approval for the version to be published.
